# Hydrogels: Characteristics and Application as Delivery Systems of Phenolic and Aroma Compounds

**DOI:** 10.3390/foods10061252

**Published:** 2021-05-31

**Authors:** Ina Ćorković, Anita Pichler, Josip Šimunović, Mirela Kopjar

**Affiliations:** 1 Faculty of Food Technology, Josip Juraj Strossmayer University, F. Kuhača 18, 31000 Osijek, Croatia; icorkovic@ptfos.hr (I.Ć.); anita.pichler@ptfos.hr (A.P.); 2 Department of Food, Bioprocessing and Nutrition Sciences, North Carolina State University, Raleigh, NC 27695, USA; simun@ncsu.edu

**Keywords:** hydrogels, edible polymers, phenolic compounds, aroma compounds

## Abstract

Complex challenges are facing the food industry as it develops novel and innovative products for the consumer marketplace. Food processing and preservation are primarily based on achievement and maintenance of safety in order to protect consumers, as well as extending product shelf life under the relevant conditions of storage, transport and distribution. Maximizing retention of bioactives with recognized positive effects on health typically comes under consideration when the previous two priorities have been achieved. This review introduces the potential applications of hydrogels as delivery systems of high-value bioactives like phenolics and aromas. If they are successfully encapsulated within the gel structures, their release can be controlled, which opens a wide range of applications, not only in food, but also in the pharmaceutical and cosmetic industries. Hydrogels are three-dimensional network structures which can absorb significant amounts of water. They have the ability to thicken the system and therefore can be used to design products with desired properties. In order to preserve the valuable components, it is necessary to know their physicochemical properties, in addition to the properties of the polymer used for hydrogel preparation.

## 1. Introduction

In food and biomaterial sciences, term gel is used to describe polymeric cross-linked network structures. Such a polymer network can be quite useful as a delivery system of various active compounds. Hydrogels are defined as three-dimensional network structures gained by natural and/or synthetic polymers that can absorb large amounts of water [1]. These swelling properties, combined with the high versatility of this material’s properties, have resulted in heightened interest in hydrogel research [2]. Hydrogels have an ability to thicken the system and in this modify texture of the products. In the food industry, they are also used to stabilize dispersed systems such as emulsions [3]. Traditional applications of hydrogels are in jellies, jams, sweets and coatings [3], but they can also be used in the preparation of gluten free products [4], as well as to extend the shelf-life of baking products [5,6]. In the literature, the word *hydrogel* was first mentioned in 1894. It was not a hydrogel as we know it today, but a colloidal gel formed with inorganic salt. There have been three main stages in the history of hydrogel developments. The first stage included developing a material with high swelling and good mechanical properties with a simple structure. Then, in the seventies, another theory was developed: hydrogels were capable of responding to environmental conditions (change of temperature, pH or concentration) by initiating polymerization of the material. The third stage of hydrogel developments was targeted on the investigation and development of complex materials. This progress has led to an increasing interest in the development of “smart hydrogels” or polymeric matrices with a wide range of properties such as the loading, retaining and releasing of fluids. These properties can be adjusted and used in perfume delivery, production of watering beads for plants, drug delivery, the food industry or cosmetics [2]. Currently, gel-like structures are used for the encapsulation of bioactive ingredients and aroma compounds whose release may then be controlled. These components are unstable but highly valuable due to their positive effect on human health. In this encapsulated form, they can be used in the preparation of innovative foods [3]. In addition to carotenoids, tocopherols and phytosterols, bioactive compounds commonly used for encapsulation with edible polymers are phenolic compounds. They are used as additives in functional foods due to their bioactivity. However, food processing affects their stability and thus reduces value of the final product [7,8]. Phenolic compounds are incorporated into films, coatings or other physical forms of hydrogel, and in this way, undesirable oxidative reactions can be reduced. Quality of the food products is affected by oxidative reactions, which can cause change of color, development of rancidity and changes in nutritional value [9]. Food quality is also affected by aroma, which is an important organoleptic property. It usually has a major influence on the acceptability and value of foods. Hydrocolloids in food matrices can influence aroma, and thus their interactions are an important subject of contemporary research [10]. Aroma compounds have several positive effects on human health [11]. Hydrogels can also play an important role in the preparation of new, edible and bioactive packaging that may reduce global plastic consumption, while at the same time meeting both market and food technology requirements [12]. Hydrocolloids are also used as substitutes for lipids in food products. Interest in hydrocolloid research has been growing due to increased awareness of the importance of a healthy diet [13]. To retain properties of a product when lipids are removed, lipid-substitutes such as starch, pectin and carrageenans are used by the industry [14]. 

Gels for food applications are usually based on polymers that are present in nature because of the need to be safe for consumption [15]. They are used in food packaging due to their ability to control the product humidity or provide antimicrobial activity [16]. They have different rheological and textural properties and therefore their applications in the pharmaceutical, cosmetic and food industries are numerous [17]. Generally, food packaging ensures food safety and prolongs its shelf life. Plastic materials have good mechanical strength and flexibility. However, these materials have undesirable non-biodegradable properties so there is a need for their replacement. Films from natural biopolymers could ensure the required anaerobic conditions, while at the same time having desirable biodegradable properties [18]. The use of edible polymers in food packaging provides a wide range of possibilities. Different bioactive compounds with positive antimicrobial and antioxidant activity can be added to the composition of films and thus improve their properties [12]. There are various studies in the literature that have studied the appropriate composition of biodegradable food films loaded with bioactive compounds [9,16,19,20,21,22]. Also, synthetic polymers used in agriculture contribute to the overall problem of environmental pollution. The application of natural polymers could reduce negative consequences of their use. There is a wide spectrum of possible applications of hydrogels such as food packaging, cosmetics, water treatment, tissue engineering, drug delivery, DNA delivery, photodynamic therapy, cell culture, wound healing, biosensors, biomedical devices and others [18].

**Figure 1:**
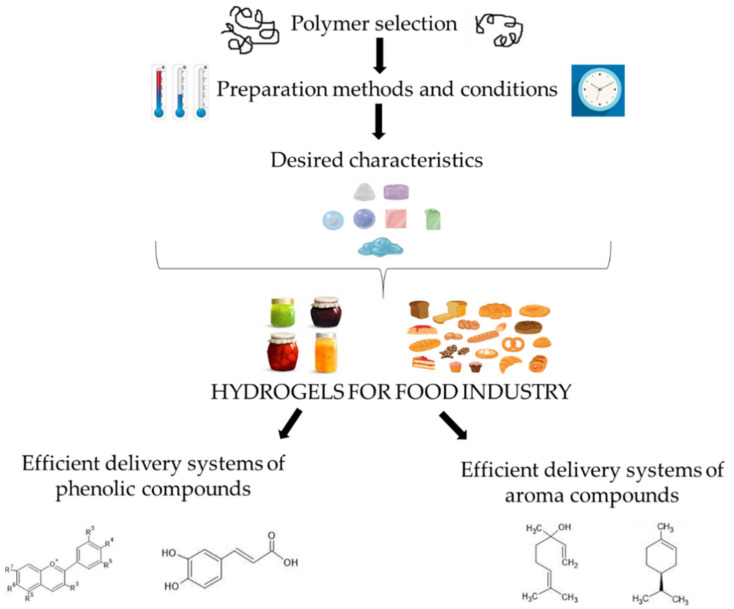
Relationship between different factors in order to obtain hydrogels as efficient delivery systems of phenolic and volatile compounds.

Looking forward, the main goal should be the creation of hydrogels which are economical to produce and safe for consumption, so that their cost/benefit ratio is improved [15]. Recently, more attention has been given to hydrogels as delivery systems of different active ingredients. Successful formulation of hydrogels as efficient delivery systems requires proper and adequate selection of polymers, preparation methods and conditions during preparation. Those factors are essential in defining the characteristics of hydrogels, and consequently their potential application in the food industry. Firstly, in this review, a summary of general hydrogel classification, their preparation methods and properties was given. An emphasis of the second part was on the application of hydrogels as delivery systems of phenolic and aroma compounds. The relationship between different factors in order to obtain hydrogels as efficient delivery systems of phenolic and volatile compounds is given in Figure 1.

## 2. Classification of Hydrogels

A wide range of water-soluble polymers with different physical and chemical properties is used to produce hydrogels [23]. The majority of phenolic and volatile compounds are quite unstable under different environmental and processing conditions; thus, their stabilization is necessary and can be achieved throughout entrapment in a hydrogel network. This is the reason why the selection of an adequate polymer is essential. Polymer properties define not only interactions during the hydrogel formation, but also their interactions with phenolic and volatile compounds in order to formulate stable hydrogels. In some cases, to ensure better entrapment of phenolic and volatile compounds, as well as their stability over time under different conditions, a combination of polymers is needed. Nowadays, utilization of natural sources is on the rise, and consequently natural polymers have an advantage over synthetic ones for hydrogel preparation. In this section, the classification of hydrogels based on their nature i.e., interactions involved in cross-linking of hydrogels and origin of polymers will be discussed. Also, the most common polymer-based hydrogels will be described. 

### 2.1. Nature of Hydrogels

Based on their nature, cross-linked hydrogels are classified into two categories: permanent or chemical gels and reversible or physical gels. Hydrophobic forces and hydrogen bonding play the main role in formation of reversible or physical gels. If environmental conditions (temperature, pH and ionic strength) are adjusted in a specific way, physical gels are dissolved [23]. They are not homogeneous due to the creation of clusters of molecular entanglements [2]. On the other hand, if covalent cross-linking takes place, formed hydrogels are called permanent or chemical, and their covalent bonding is made more stable, as well as stronger [1]. Chemical gels are developed when hydrophobic polymers are converted to hydrophilic through the cross-linking of water-soluble polymers [2]. 

### 2.2. Origin of Hydrogels

Based on the origin, hydrogels can be classified into two categories: natural and synthetic [24]. Natural hydrogels are derived from animal and plant macromolecules. They are known for their safe usage, low toxicity, biocompatibility and biodegradability. Commonly used natural polymers are collagen, hyaluronic acid, fibrin and derivatives of natural materials such as starch, alginate, chitosan and skill fibers. There are two main disadvantages of using natural polymers which make hydrogel’s final microstructures and properties difficult to control. Firstly, polymers’ mechanical properties and their dependence on conditions of polymerization or gelation can sometimes be difficult to explain. Secondly, their composition varies from one batch to another due to their natural origin. Often it is impossible to obtain the identical composition of desired products [2]. On the other hand, synthetic hydrogels are usually not biodegradable and can develop toxicity from trace chemicals [25]. Often used synthetic polymers are acrylic acid-hydroxyethyl methacrylate, vinyl acetate and methacrylic acid [26]. However, using synthetic hydrogels also has some advantages since they have a precise chemical structure and can be re-designed with the purpose of making new, environmentally friendly hydrogels [25]. In general, synthetic hydrogels provide a greater flexibility to adapt chemical composition and mechanical properties by changing the amount of crosslinkers or varying the initial concentration or molecular weight of the precursor [2]. Lower animals and humans can consume edible polymers without any harmful effects and thus the Food and Drug Administration has regarded edible polymers as safe (GRAS). Edible polymers are hydrocolloids (starch, hydroxypropyl cellulose, alginates, gums, carrageenan), proteins (animal based: gelatine, collagen, albumin; plant based: zein, soy, wheat gluten) and lipids (fatty acids, triglycerides, phospholipids) [18].

These edible polymers are used as substitutes for synthetic plastic because they can be consumed like food itself. Hydrogels based on polysaccharides have properties of the highest quality thanks to their biocompatibility and adjustable functionalities which can be applied in the food industry, but also in bioengineering, drug delivery, etc. Global pollution and environmental contamination are one of the major issues that the human population is facing. There are six different physical forms of hydrogels: liquids, microparticles, coatings, solid molded forms, membrane/films and pressed powder matrices [18].

### 2.3. Hydrocolloid-Based Hydrogels

#### 2.3.1. Chitosan-Based Hydrogels 

Chitosan is a polysaccharide formed by deacetylation of chitin. It has been the subject of contemporary research efforts due to its low toxicity and biocompatibility. It can be modified into different shapes (hydrogels, beads, films, membranes and powders) and then used in various areas, such as food engineering, pharmaceutical industry and medicine. Usually, chitosan hydrogels are formed by using the sol-gel method in which chitosan is dissolved in acid. After complete dissolution, cross-linkers are joined together and polymer chains are covalently bonded, thus forming the permanent hydrogel network. Chitosan hydrogels are improved by chemical or physical modifications, and the selection is made according to the desired characteristics of the final product (e.g., porosity, swelling degree, its ability to maintain aroma or release fragrance) [27].

#### 2.3.2. Pectin-Based Hydrogels 

Pectin is a polysaccharide obtained from the primary cell wall of apples and citrus fruits [28]. Its chemical structure consists of “smooth” and “hairy” regions. “Smooth” regions are partially methylated poly-α-(1→4)-D-galacturonic acid residues called galacturonan. “Hairy” regions are heteropolymeric α-(1→2)-L-rhamnosy-α-(1→4)-D-galacturonosyl sections which contain branch-points with mostly neutral side chains of mainly L-arabinose and D-galactose (rhamnogalacturonan I). Pectin may also contain rhamnogalacturonan II with sidechains containing residues other than arabinose and galactose. These sidechains are made of D-xylose, L-fucose, D-glucuronic acid, 3-deoxy-D-manno-2-octulosonic acid, D-apiose and 3-deoxy-D-lyxo-2-heptulosonic acid attached to poly-α-(1→4)-D-galacturonic acid regions [25]. Pectin can be categorized from low-methoxyl to high-methoxyl based on its esterification degree. By lowering pH or application of divalent or trivalent ions, the polymer network can be formed [2]. An important characteristic of pectin with low esterification degree is the formation of coacervate in the presence of Ca^2+^ ions. This is used for controlled release of important aromas, proteins or other bioactive compounds mainly in the food and pharmaceutical industries; also, it helps to accomplish the desired firmness of food products [25]. High-methoxyl pectin is used in the preparation of cloudy juices, beverages, ice creams, mayonnaise, tomato ketchup and jellied sweets. Pectin is also a member of the “roughage” group, which is known for its positive impact on human health, such as the reduction of cholesterol levels in blood [29].

Figueroa and Genovese [30] prepared pectin hydrogels enriched with different types of dietary fiber. Apple, bamboo, wheat and psyllium fibers were used for the determination of mechanical properties (hardness, fracturability, cohesiveness) and water loss with the aim of obtaining new types of enriched jams. Samples with psyllium fiber were gummy, apple fiber caused change of color, while wheat and bamboo fiber created floury mouthfeel of prepared jams. Due to the negative effects on mechanical properties, authors mixed 0.5% pectin, 1.5% psyllium and 1.5% of other fibers, so gels with good mechanical properties were obtained.

Xie et al. [31] demonstrated the application of hydrogel microspheres which were formed from gelatine and pectin as fat and starch replacements. These hydrogel microspheres can be formulated using electrostatic complexation. Association of gelatine and pectin molecules with each other occurred through electrostatic attraction by changing the solution conditions. This leads to separation of phases, and thus a polymer-rich phase and a polymer-depleted phase are formed. Particles have been promising candidates for development of healthy, reduced-calorie foods due to the fact that they were formed using proteins and dietary fibers. The complexation which occurs during reduction of the pH of gelatine and pectin includes several stages. Firstly, gelatine and pectin are separated molecules due to the electrostatic repulsion. Positive parts of gelatine and negative parts of pectin are attracted because of different electrostatic charges between them. After that, soluble complexes join and form gelatine-pectin sub-units which are linked together, forming hydrogel particles. At the end, when temperature is lowered, internal structure sets [31].

#### 2.3.3. Alginate-Based Hydrogels 

Alginate is a polysaccharide obtained from brown seaweed. Its chemical structure consists of linear 1→4-linked glycuronans which are composed of β-D-mannopyranuronic acid (M) residues and α-L-gulupyranuronic acid residues (G). Both residues can form homopolymeric (GG or MM) and heteropolymeric (GM) blocks. As for pectin, multivalent cations (like calcium, magnesium, manganese and aluminium) are used for alginate cross-linking [25]. The resulting binding strength follows the order: Cu^2+^ > Co^2+^ > Zn^2+^ > Mn^2+^ ≫ Mg^2+^ > Ca^2+^ > Sr^2+^. Alginate hydrogels are tough and thus used in biomedicine for the delivery of unstable compounds [18]. If exposed to cations listed above, alginate chains interact with the ions, and cross-linking with the nearby chains occurs. With increasing concentration of Ca^2+^ ions, extent of cross-linking and chain immobilization also increases. This enhances hydrophobic interactions in the sample, which were illustrated by Da Silva Fernandes and co-workers [32].

#### 2.3.4. Carrageenan-Based Hydrogels 

According to its chemical composition, carrageenan is a sulphated polysaccharide obtained from red algae [33]. It is mainly used in the dairy industry because of its thickening and stabilizing properties. According to the number and position of sulphonic groups which are present in their structure, carrageenans are categorized as: kappa (κ), lambda (λ), iota (ι), mu (μ), nu (ν), and theta (θ), where kappa, lambda and iota are among the most used ones [18]. The coil-helix transition followed by aggregation of double helices makes up the gelation process of κ-carrageenan. The formation process of carrageenan hydrogels depends on polymer concentration, the presence of counter ions and the ratio and the position of sulphonic groups. Κ-carrageenan forms hard, brittle and strong hydrogels, promoted by the monovalent cations, and ι-carrageenan forms weak and soft hydrogels promoted by the presence of divalent cations [34]. In the case of κ-carrageenan, the ability of cations to enhance the formation of gels increases as follows: non-specific monovalent cations (Li^+^, Na^+^), divalent cations (Ca^2+^, Mg^2+^) and specific monovalent cations (K^+^, Cs^+^) [35]. Textural differences of hydrogels prepared from different types of carrageenan are the result of different gelation processes. In the case of κ and ι carrageenan, gelation is initiated by the anhydro-galactose bridge in the 4-linked galactose residue [34]. Smaller monovalent cations (Li^+^, Na^+^) form only ionic bonds and thus reduce their ability to control the flexibility of disaccharide units of carrageenan [36]. Cooling triggers the creation of quaternary structures which are formed by intramolecular Ca^2+^ bridging. Monovalent cations such as K^+^ induce intermolecular associations by creating ionic bonds between sulphonic groups and K^+^. Anhydro-bridge oxygen atom of adjoining galactose residue and K^+^ form secondary electrostatic bonds. The absence of this bridge in λ-carrageenan prevents the formation of hydrogels. Still, it forms highly viscous systems and thus can be used as a thickener [34]. As a result of its biocompatible and biodegradable properties, it is widely used in food industry as a texturing agent [37]. Zheng et al. [38] prepared food-grade bigels from κ-carrageenan and monoglycerides for delivery of β-carotene. It was concluded that bigels increased β-carotene stability and in-vitro gastrointestinal release. For that reason, new hydrogels based on two or more edible polymers have been developed [39]. Addition of sucrose also improves strength and stability of carrageenan gel network [37]. Nakagawa et al. [40] prepared ternary system of chitosan, κ-carrageenan and CMC sodium salt for the delivery of curcumin, and the system was reported to be an efficient carrier of selected bioactives. Soares et al. [39] prepared hydrogels from κ-carrageenan and galactomannan. The results obtained in this study showed that hydrogels were physically stable and microbiologically correct during the 90-day storage and could be used in drug delivery applications, pharmaceuticals and cosmetics.

#### 2.3.5. Starch-Based Hydrogels

Starch is a polysaccharide with plant origin. It is stored in plant’s seeds, roots and in the endosperm of cereal’s grain. It serves as an important source of energy for humans (4 cal per gram) and by its hydrolysis, glucose is obtained. These long-chain glucose polymers from which starch granules are composed are insoluble in water. If stirred, starch granules form a suspension in which they absorb water and swell slightly. Once starch is cooked, its granules are broken because of the irreversible swelling. Therefore, it is used as a thickener in the food industry [41]. Properties of starch-based hydrogels are like plastic; they are tasteless, odorless, semipermeable to carbon dioxide and nontoxic, which makes them useful for food packaging, drug delivery and biomedicine [18]. Depending on the starch source, different starch gel structures can be obtained [42]. In the study of Zhang et al. [43] starches from different sources (wheat, corn, tapioca, sweet potato and potato) were investigated and it was concluded that there are significant differences in starch strength depending on the source. Decrease of network stability and resistance to deformation were in the following order: potato, sweet potato, tapioca, corn and wheat starch. These differences arise as a result of different native properties of the starches. In the potato starch, absence of lipids and presence of high phosphate monoester content are probably responsible for high strength and stability and phospholipids may be responsible for the reduction of strength in corn starch [44]. Except for origin, addition of other hydrocolloids (guar, xanthan gum, carboxymethylcellulose or hydroxypropylmethylcellulose) can also modify gelation properties of starch [45].

Starch hydrolysates are also important ingredients in the food industry and are usually characterized by dextrose equivalent value. This value describes the total reducing power of all sugars expressed by dry weight, where 100 represents the reducing power of glucose. Hydrolyzation of starch with dextrose equivalent below 20 results in the formation of maltodextrins. Maltodextrins contain D-glucose, maltose, oligosaccharides and polysaccharides. They are water soluble, as opposed to the native starch. Interactions between amylose molecules and amylopectin chains are responsible for network formation or gelation. Due to their diverse properties and complexity, maltodextrin-based gels can be used to adjust properties of food products and their quality [46].

#### 2.3.6. Cellulose-Based Hydrogels 

Cellulose is a polymer containing at least 3000 glucose molecules joined by β-1, 4-glycosidic bonds. Both cellulose and hemicellulose are not digested in the body and thus do not provide energy for humans. Still, they provide insoluble dietary fiber without which a balanced diet cannot be properly maintained [41]. Cellulose has biocompatible properties and its derivates like carboxymethylcellulose (CMC), methylcellulose (MS), hydroxypropylcellulose (HPC) and hydroxypropylmethylcellulose (HPMC) are also used as edible polymers [18]. CMC is also called the cellulose gum. It increases the viscosity of foods and is used as a binder and thickener in food products like puddings or pie fillings. If CMC is added, it gives the bulk mouthfeel, which is usually achieved by using sucrose, so CMC can be used as a replacement for sucrose in dietetic foods [41]. HPMC has an application in the food industry as the main hydrocolloid used as a strengthening agent in gluten free bakery products [4,47] or for stabilizing frozen bakery breads [6]. Cellulose hydrogels can also be used for the dewatering of fruit juices [48].

### 2.4. Protein-Based and Lipid-Based Hydrogels 

Gelatine is a heterogeneous mixture of peptides obtained by the hydrolysis of collagen. It is easily biodegradable and has great biocompatible properties. Therefore, it is used in the pharmaceutical, cosmetic and food industries. Even under low temperature conditions, gelatine hydrogels are formed and upon heating, hydrogen bonds are broken which makes gelatine hydrogels reversible [18,49]. However, gelatine gels with low strength have limited applications. Hydrogen bonding between and within the molecules, triple-helix content and molar mass distribution of gelatine affect the properties of its gels [50]. Whey proteins also have an ability to form gels. However, if pH value is above its isoelectric point, their ability to swell is very sensitive and their range of industrial application is very narrow [18]. Although protein-based hydrogels are commonly used, their stability can be improved when they are combined with polysaccharides. Its network protects proteins from external conditions, such as an acidic environment in the stomach [51].

Plant proteins have a wide range of applications in the food industry due to their ability to increase food value and change physical and colloidal properties of the system. Recently, popularity of a plant-based diet is growing and consumer interest in animal-based proteins is declining. Significant sources of plant proteins are legume seeds, zein obtained from corn kernels, and soy proteins from soybean and wheat gliadin. These are used in the food industry for their low price, high availability, nutritional value and positive health effects [51]. Lipids are usually combined with polysaccharides whenever they are used as edible polymers. Examples of such lipids are natural wax and monoglycerides with lipid character [52].

### 2.5. Hydrogels According to Their Preparation

Based on the process of preparation, hydrogels can be homopolymers, copolymers, semi-interpenetrating networks and interpenetrating networks. Homopolymers are built of single units of monomer [26]. According to the nature of the monomers and the method of their polymerization, homopolymers can have a cross-linked skeletal structure [24]. Two or more different monomers, where at least one is hydrophilic, can form copolymeric hydrogels. Linear polymers, which penetrates another cross-linked network without any chemical bond, are semi-interpenetrating networks. On the other hand, an interpenetrating network is a combination of two polymers where one of the polymers is synthesized in the presence of the other [26].

## 3. Methods of Hydrogels Preparation

Generally, different types of methods exist for the preparation of hydrogels. Each method is characterized by a specific mechanism and conditions. One factor that is important for the utilization of certain methods for the preparation of hydrogels is already mentioned physical and chemical properties of polymers. When hydrogels are used as delivery systems of phenolic and volatile compounds, behavior of these compounds under conditions of the selected method are crucial. Both phenolic and volatile compounds can undergo different chemical reactions such as oxidation, polymerization, condensation and degradation, which lead not only to the change of their chemical structures but also to the change of their functional properties. In order to prepare an efficient hydrogel, those negative alterations have to be minimized.

Forming a larger branched polymer by linking the macromolecular chains together is called the gelation process. The formed mixture is called “sol” and further linking results in a mixture called gel. The transition from finite to infinite structure is the “sol-gel” transition. The point in which this transition happens is “gel point” [1]. 

### 3.1. Physical Cross-Linking 

Physical gels are reversible and easy to produce, and thus the interest in their production has grown. Cross-linking agents are not required in this process and the structure of high-value substances, which we want to deliver by forming a hydrogel, is not affected (e.g., cells, proteins) [26]. Also, the absence of cross-linking agents lowers the hydrogel toxicity and enhances its biocompatibility [18].

#### 3.1.1. Heating and Cooling 

An example of this cross-linking technique is the preparation of carrageenan hydrogels. By heating its solution above the melting point, it is present as a random coil conformation and when cooled it is transformed into a rigid helical rod. If salt cations are present (Na^+^ or K^+^), repulsion of sulphonic (H-SO_3_^−^) group will follow and double helices will aggregate to form stable gels [26]. Ι-carrageenans form soft elastic gels in hot and cold water, while κ-carrageenans form strong gels only in hot water. Polymers with hydrophobic groups can cross-link in an aqueous environment via “sol-gel” transition. These interactions take place at elevated temperatures in solutions of amphiphilic polymers. As temperature increases, hydrophobic domains aggregate and minimize the contact with water molecules, which causes the solvent entropy to reach its maximum. If this segment is larger, solvent entropy is higher and gelation temperature is lower [18]. At pH values below 4.3, viscosity and gel strength of carrageenan gels are reduced [53]. Another example of gel obtained after heating and cooling is starch gel [54]. After heating an aqueous starch suspension above gelatinization temperature, swelling of starch granules occurs and sudden cooling converts the fluid into a turbid viscoelastic paste. Opaque elastic gel is formed at starch concentration above 6410 *w*/*w* [55].

#### 3.1.2. Complex Coacervation

Blending of polymers with opposite charges, depending on external factors (pH and concentration), can result in the formation of soluble or insoluble complexes. An example of this method is coacervation of polyanionic xanthan and polycationic chitosan [26]. In nature, microorganisms *Xanthomonas campestris* produce polysaccharide xanthan gum while in the industrial processes it is produced by fermentation. Xanthan gum has an ability to thicken and stabilize food systems and therefore it is used as a food additive [56]. At low shear rates, xanthan gum is very viscous, but at high shear rates it has very low viscosity. It is a pseudo-plastic behavior stabilizer which is soluble at low temperatures. Due to its ability to tolerate a wide range of acidity and heat levels, it is often used as a stabilizer for sauces and dressings in the food industry [53]. As mentioned earlier, chitosan is derived from chitin and used in the food industry for its chelating properties in order to remove unnecessary and potentially harmful elements and particles [57].

#### 3.1.3. Ionic Interaction 

Addition of di- or tri-valent counterions encourages the formation of ionic polymers. The principle of this method is the same as the one for gelling a polyelectrolyte solution with oppositely charged ions (e.g., Na^+^ alginate- and Ca^2+^ + 2Cl^−^) [26]. Hydrogels formed by this type of interaction are usually cross-linked under mild conditions (physiological pH and ambient temperature). Also, gelation rate in this process is difficult to control and gels have non-uniform structures [18].

#### 3.1.4. Freeze Thawing

Hydrogels can be formed by freezing a solution of polymers at low temperatures (from −20 °C to −80 °C), which is followed by melting at room temperature. Their properties can be modified by changing pH, temperature and freezing duration [18]. Using this method, Guan et al. [58] prepared hydrogels with high mechanical strength and thermal stability from hemicellulose, polyvinyl alcohol and chitin.

### 3.2. Chemical Cross-Linking 

To link two polymer chains using chemical cross-linking, either the monomers are grafted on the polymers or cross-linking agents are used. This linking is accomplished by the reaction between functional groups (e.g., OH, COOH and NH_2_) with cross-linkers (e.g., aldehydes like glutaraldehyde) [26]. Unlike physical cross-linking, hydrogels formed by chemical cross-linking are mechanically strong and resistant to the dilution of matrix. In this type of hydrogel preparation, a new molecule is inserted between polymer chains. However, some types of cross-linkers like glutaraldehyde, glyoxal and epichlorohydrin can increase the toxicity of hydrogels [18].

### 3.3. Grafting Cross-Linking 

Grafting is the addition of a monomer on the backbone of a polymer and this process starts with the activation of polymer chains by high-energy radiation treatment or by chemical reagents [18]. Hydrogels made through bulk polymerization usually are characterized by weak structure, so in order to improve these weak mechanical properties, they are grafted on a surface which is coated onto a stronger support. Throughout this process, free radicals are generated onto a stronger support surface and then polymerizing monomers are covalently bonded onto it [24].

### 3.4. Polymerization through Irradiation

Gamma rays, electron beams or different high-energy radiation types are used to start the hydrogel preparation of unsaturated compounds [24]. According to the method used for the preparation, hydrogel particles have different forms which affect rheological properties and retention rates. Also, different shapes and structures of hydrogels can be prepared, such as spheres, ellipsoids, disks, needles, homogenous, core-shells and dispersion [59].

It is important to note that all these preparation methods can be transformed from laboratory to large-scale production and that they are safe and non-toxic for a wide range of products. Gums with natural origin are biodegradable, bioavailable, accessible and nontoxic, and thus have an advantage for encapsulation of sensitive ingredients and their economical production in the food industry [53].

## 4. Characteristics of Hydrogels

Hydrogel characteristics are result of polymer properties and preparation method. They define utilization of hydrogels, especially when hydrogels are used as functional additives. Also, characteristics of hydrogels can affect stability of phenolic and volatile compounds over time and under different conditions, as well as their release in the product. Obtaining hydrogels of desired characteristic ensure preparation of efficient delivery systems which can improve products quality. Both phenolic and volatile compounds delivered into the product can improve antioxidant stability of the product, nutritional value, color and aroma. Also, many of phenolics and volatiles have an antimicrobial effect, and thus hydrogels can be used to prolong a product’s shelf-life. This section describes the most important hydrogel characteristics.

### 4.1. Swelling 

One of the most important characteristics of hydrogels is swelling. Polymer network of hydrogels can absorb 10 to 1000 times of their dry weight in water. An obtained network holds the system together while absorbed liquid is used as a selective diffusion filter [23]. In contact with water, polar groups are hydrated and this results in formation of primary bound water. The next step is the formation of secondary bound water or hydrophobically bound water, when hydrophobic groups interact with water. Primary and secondary bound water add up to the total bound water. Osmotic force leads to the absorption of the rest of the water called free water, which fills the space between the networks [1]. Swelling is determined by measuring the dry weight and weight of the hydrogel in a swollen state. Determination of this property is very important because other properties like the cross-linking degree or the degradation rate are derived from this value. Water uptake (1) and volume of adsorbed solvent (2) can be calculated and expressed in percentages [23].

(1) $$\mathrm{Water}\;\mathrm{uptake}=\frac{\mathrm{swollen}\;\mathrm{weight}\;-\;\mathrm{dry}\;\mathrm{weight}}{\mathrm{dry}\;\mathrm{weight}}\;*\;100$$

(2) $$\mathrm{Volume}\;\mathrm{of}\;\mathrm{adsorbed}\;\mathrm{solvent}=\;\frac{\mathrm{swollen}\;\mathrm{weight}\;-\;\mathrm{dry}\;\mathrm{weight}\;}{\mathrm{water}\;\mathrm{density}}\;*\;100$$

### 4.2. Mechanical and Rheological Properties 

Variation of the cross-linking degree affects the stiffness of hydrogels. Data on the mechanical properties (Young modulus or storage and loss moduli) is obtained by using a rheometer and dynamic mechanical analysis [23].

Based on the physical structure of a biopolymer network, hydrogels can be categorized as strong, weak or pseudo gels. Different textures arise due to the different hydrocolloids used in the production of gels. Xanthan and gellan gum form soft and flexible gels, starch, alginate, pectin, gelatin, ι-carrageenan form a typical gel texture while firm, brittle gels are formed from agar and κ-carrageenan. Rheological measurements are a useful tool in characterizing the gelling degree of polysaccharide solutions [60]. At low solids content, hydrogels are polymer solutions with increased viscosity and if shear stress is applied, they can flow. However, hydrogels also have the characteristics of hard-sphere suspensions. Whenever the solvent composition is modified by increasing the volume of the gels in the suspension or by changing its pH value, polymers change their conformation. Rheology of hydrogels depends on the intensity of interactions between particles and the resulting network structure. In addition, properties of hydrogels are modified if their conformation changes (e.g., degree of cross-linking, particle size, size distribution, particle shape). Proper manipulation can affect the hydrogel’s characteristics in a way that obtained hydrogels meet some industrial requirements and thus improve stability or prolong shelf life of foods. Hydrogel suspensions generally do not have non-linear viscoelastic properties, which are unpleasant in some food systems like dressings and beverages, so they can be used as an alternative [15].

Garzon et al. [42] characterized gels from corn, rice, wheat and potato starch using a rapid force analyzer and changes in gels were observed during 90 s of heating and stirring. It was concluded that time to reach gelatinization varies according to starch sources and that parameters obtained from rapid force analyzer were in correlation with gelatinization temperature. The highest force was detected for potato starch followed by wheat starch, while corn and rice starch had no significant difference.

Rosell et al. [61] investigated the effects that different hydrocolloids and its combinations (HPMC, pectin, guar and xanthan gum) have on mechanical properties of wheat dough after thermal treatment. Dough is a viscoelastic material and has rheological behavior between a viscous liquid and elastic solid. During heating, changes in rheological properties occur. HPMC caused greater reduction of dough consistence. The addition of guar gum can offset this negative impact of HPMC, and when added independently it reduces the weakening of the dough. Guar gum gives the wheat dough a certain strength to resist mechanical shear stress during overmixing. Pectin and xanthan gum did not show the significant effect on the mechanical weakening when added singly, only when added in the combination with guar gum or HPMC. These results proved the importance of interactions between polymers and their effect on hydrogels characteristics.

The total stress for viscoelastic materials can be divided into the in-phase and out of phase components. Applied strain divides the stress to determine storage (G′) and loss (G″) modulus. Storage modulus defines the solid-like (elastic) behavior of the sample and the loss modulus defines the liquid-like behavior. Relation of G′ and G″ with frequency, normally shown on logarithmic axes, are known as the “mechanical spectrum” and η* shows the complex viscosity and is usually shown in spectrum. The typical mechanical spectra was illustrated by Zhang et al. [60]. They showed that in strong polysaccharide gels, solid-like behavior (G′) predominates over liquid-like (G″) with slight changes in modulus on varying frequency and log η* decreases linearly as log ω increases. In weak polysaccharide gels, elastic and viscous moduli frequency dependence is limited. Mechanical spectrum of pseudo gels or fluid gels showed strong dependence of G′ and G″ on frequency. On the other hand, mechanical spectrum of dilute solutions had G″ higher than G′ across the entire frequency range [60]. Rosell et al. [54] studied the influence of different hydrocolloids (HPMC, guar and xanthan gum) on dynamic rheological properties of rice starch. The starch paste and combination of different hydrocolloids and starch had G′ > G″ that is typical for gel behavior. The storage and loss moduli decreased when starch concentration was reduced. However, mechanical spectrum of G″ was considerably more affected than spectrum of G′. Increase of G′ was caused by addition of guar and xanthan gum. The smallest effect on G′ was observed for HPMC. This behavior was explained by the network structure derived by the reduced number of junction zones of the starch when in combination with entanglements of hydrocolloids. It was concluded that different hydrocolloids affected the rheology of the rice starch paste.

### 4.3. Porosity and Permeability

Pores are formed during the process of phase separation. Tortuosity is a parameter described by the average pore size and pore interconnections. It has been found that 3 main factors affect pore size:

- Ratio of monomer concentration to concentration of cross-linker
- Concentration of polymerizable monomers present in the system
- Concentration of cationic and/or anionic monomer.

Porosity of a hydrogel is affected by the composition of the solution in the environment by Gibbs-Donnan and osmotic effects [23].

Gehrke et al. [62] studied factors determining hydrogel’s permeability. They concluded that there is a direct relationship between swelling properties of hydrogels and permeability of solute. Many hydrogel-based delivery systems use hydrogels as coatings, capsules and membranes. If the water content in the gel decreases and swelling of the gel is reduced, the mobility of solutes is limited because the average pore size of the gel network is reduced. Permeability is the result of transport properties (diffusion coefficient D_i_) and the thermodynamic properties (partition coefficient K_i_) and i denotes the steady state flux of solute across a membrane from a donor phase to a receptor phase:

(3) $$j_{i\;}=\;\frac{K_{i}*\;D_{i}}{L}{\;(C}_{\mathrm{di}}-C_{\mathrm{ri}})$$

where j_i_ (3) is flux of solute i (mol/cm^2^·s), C_di_ is concentration of solute i in donor phase (mol/cm^3^), C_ri_ represents concentration of solute i in receptor phase (mol/cm^3^), L is membrane thickness (cm) and D_i_ is diffusion coefficient of solute i (cm^2^/s). K_i_ (4) represents the partition coefficient of solute i (dimensionless) and is expressed as follows:

(4) $$K_{i}=\frac{C_{\mathrm{gi}}}{C_{\mathrm{si}}}$$

where C_gi_ is concentration of solute i in the gel membrane (mol/cm^3^), C_si_ is concentration of solute i in solution (mol/cm^3^). P_i_ (5) is permeability of solute i (cm^2^/s) and is expressed as follows:

(5) $$P_{i\;}{=K}_{i}{*\;D}_{i}$$

To understand hydrogel permeability, it is necessary to know how gel network affects the transport and also the thermodynamic properties of the system. Fick’s First Law (6) defines flux (j_i_) as the product of the diffusion coefficient (D_i_) and the concentration gradient (dC_i_/dz):

(6) $$j_{i}=-D_{i}*\;\left(\frac{{\mathrm{dC}}_{i}}{\mathrm{dz}}\right)$$

D_i_ value depends on molecular weight of solutes (from values on the order of 10^−5^ cm^2^/s for simple organic molecules to values on the order of 10^−7^ cm^2^/s for proteins). Thermodynamic interactions between the solute and the network also lead to alterations in the solute concentrations, and thus K_i_ is changed. The presence of attractive interactions (e.g., electrostatic interactions) results in increased K_i_ value which consequently increases the value of permeability. An additional property which affects permeability is gel’s heterogeneity. It is of great importance to know where within the gel the solute is diffusing. Finally, nature of the water within the gel (free or bounded water) also has an impact on the solute diffusions in gels. Solute transport does not occur through membranes in the absence of free water [62].

### 4.4. Cross-Linking

All other properties of hydrogels are the result of cross-linking. By changing its degree, other properties can be adjusted and just one type of polymer can be used for different applications. There are two types of cross-linking; physical which is encouraged when hydrophobic interactions between the chains occur, and chemical which usually begins with heating or UV radiation [23].

### 4.5. Biocompatibility

Biocompatibility means that hydrogel and its products are compatible with the human immune system. In contact with body fluids, hydrogels have low tendency for proteins and cells to adhere to their surface because of their low interfacial free energy. Modifications in their polarity, swelling behavior, mechanical and surface properties result in chemical changes as well [1].

### 4.6. Target Delivery

Encapsulation is used in the food industry for protection of sensitive bioactive components in hydrogels and for their target delivery. This process is used when it is necessary to mask unpleasant aromas or to increase satiety. High-value bioactive compounds are preserved by insertion into the polymer network [15]. Bioactive compounds from natural products are mostly used for encapsulation because they are problematic when it comes to translating therapeutic and clinical effects from in-vitro to in-vivo conditions due to their low oral absorption. They are also unstable to other external stimuli such as heat, light, oxygen and enzymes. However, in delivery systems their stability is improved because gel networks separate this component from the environment [17]. Using this process, their stability and bioavailability are improved and their release rate is easily managed. Components which enhance bioactivity of the natural material are phenolic compounds, carotenoids, tocopherols and phytosterols [7]. There is a wide range of bioactive molecules that can be encapsulated into hydrogel particles (such as solid particles, liposomes, different types of emulsions) [59].

## 5. Retention of Phenolic Compounds in Hydrogels

All previously mentioned characteristics could be adjusted with the aim of obtaining smart systems for delivery of bioactive compounds [63]. Changes of external conditions (temperature, solvent polarity, ionic strength, electric/magnetic field, light, addition of biomolecules) affect properties of hydrogels [64]. Since they have biocompatible properties, hydrogels are used as a tool for the delivery of plant-derived phenolic compounds with low toxicity and exceptional wide range of biological properties. Polyphenols represent a chemical group for which structure is known as an aromatic ring and one or more hydroxyl substituents or other functional groups like esters, glycosides etc.. Polyphenols are one of the most extensively studied plant compounds. These secondary metabolites play the main part in UV protection and rejection of microbes and herbivores in plants [63]. Phenolic compounds are known for their antimicrobial and anti-inflammatory activity and thus they have been the subject of numerous studies. Considering all these aspects, scientists want to develop hydrogels which can both retain and preserve these high-value compounds [64]. Water-soluble phenolic compounds are phenolic acids, flavonoids and quinones; water-insoluble phenolic compounds are tannins, lignins, hydroxycinnamic acids. Targeted delivery of phenolic compounds has strict limitations due to low bioavailability, bioaccessibility, solubility and stability. Bioaccessibility refers to in-vitro digestion whereas bioavailability studies the ingredients that have been actually absorbed in the gastrointestinal tract (in vivo). Undesirable odors and brown color which arise in food systems as products of the oxidation of phenolic compounds damage product quality, and thus it is important to create carriers which ensure their stability [53]. Proteins and polysaccharides can be used in combinations to formulate hydrogels. Tannic acid, caffeic acid, proanthocyanins and procyanidins are some of the natural cross-linkers used for hydrogel formation. Chemical reaction starts with oxidation of the phenolic structure and quinone is formed. It then reacts with nucleophiles of amino acid groups (e.g., sulfhydryl, amide, amine) in protein chains. Kosaraju et al. [65] used caffeic acid for the preparation of gelatine gel. Conditions required to obtain a stable gel were a cross-linking reaction at 60 °C and pH 9 for 20 min. Gel strength at high temperatures was higher for the cross-linked hydrogels than non-cross-linked [16]. Maier et al. [66] prepared pectin and gelatine gels enriched with grape pomace extracts. Antioxidant activities of the samples, total phenolic contents, anthocyanins and non-anthocyanin phenolics were determined by HPLC and spectrophotometric methods throughout the storage period. The objective of the study was to evaluate how processing, food matrix composition and storage affected phenolics, antioxidant activity and color of gels. It was concluded that the heating the mixture during the gel preparation to a temperature of up to 75 °C for 2 min certainly had an impact on the reduction of phenolic content of both gels. Gelatine gel had a loss of 24.6% of total phenolics and 79% of non-anthocyanin flavonoids, while pectin gel had a loss of 15.2% of total phenolics and decrease of non-anthocyanin of 76%. Retention of phenolic acids in prepared systems was more efficient, only 5.1% of phenolic acids were lost in pectin gels and in gelatine gels the content of phenolic acids remained constant. Authors explained this phenomenon as a stabilizing effect of pectin on anthocyanins assigned to ionic interactions which can occur between the anthocyanin cations and the carboxylic groups of pectin. Also, pectin gels had better retention of anthocyanins, probably due to the intermolecular interactions with polyuronic acid. Predominant components of non-anthocyanin flavonoids were catechin and epicatechin. This is caused by the high-molecular phenolic compounds decomposing into smaller fragments. Storage conditions affected pectin and gelatine model systems differently. One of the possible reasons for these differences is the interaction between phenolics and matrix components (e.g., proteins, polyuronic acid). Gallic and syringic acid contents were higher in pectin and gelatine stored samples because these acids are the degradation products of tannins. These results confirm the finding that polyphenol stability depends on the matrix composition [66].

Rutin, as a plant flavonoid, is a rhamnoglucoside of the flavonoid quercetin. It is found in many plants and can be used for treatment of various vascular diseases. Soni et al. [64] prepared hydrogels by dissolving polymers (hydroxypropyl methylcellulose, carbopol 934 and acaica) in small amounts of distilled water in various proportions. After that, the remaining ingredients (glycerine, sodium benzoate) and rutin (methanolic dispersion; 1 mg/mL) were added to it. Results of that study showed that the hydrogel containing 0.025% of rutin showed significant antimicrobial and anti-inflammatory effect compared to activity of hydrogels containing 0.020% and 0.030% of rutin.

Anthocyanins are known and recognized for their positive effects on human health (antioxidant activity, anti-cancer functionality). Still, they are very sensitive to the changes of pH values in their environment, so one of the main challenges in the food industry is to prepare systems for their delivery. Jin et al. [67] prepared hydrogels from konjac glucomannan and xanthan gum with the aim of anthocyanins preservation. Investigation of rheology proved that hydrogels were strongly dependant on temperature. It was concluded that stronger hydrogel network had superior protective effects on encapsulation of anthocyanins, regardless of the change in pH values.

In the study by Betz et al. [8] anthocyanins from bilberry extracts were used for encapsulation in whey protein. Loss of phenols was observed and it was concluded that it occurred due to interactions between the proteins and other bilberry compounds. Using microencapsulation, anthocyanins can be successfully delivered. Antioxidant activity was affected both by heating in gelation process and by interactions of polyphenols and proteins. Bioavailability of anthocyanins is low because they are sensitive to pH values, oxygen, temperature, presence of other solutes and UV light. For those reasons, it is important to ensure their stable delivery. In an acidic environment, anthocyanins are present in the form of flavylium cation. Due to the pH values, they are present in the same form in the stomach but in the intestine they are converted to quinonoidal, hemiketal and chalcone forms.

Najafi-Soulari et al. [68] encapsulated phenolic compounds from lemon balm known for antioxidant activity (carnosic acid, rosmarinic acid, isoquercitin and rhamnocitrin) in hydrogel beads prepared from alginate with or without chitosan layer. By dropping the alginate with a syringe into the container with calcium solution, hydrogel beads were prepared. Encapsulation did not cause changes in antioxidant activity. Addition of the chitosan layer caused the reduction of encapsulation efficiency. Beads with antioxidant properties can be used for the formation of new, enriched food products.

Aguirre Calvo et al. [69] examined the formation of alginate gels with the addition of Ca^2+^ ions and sucrose, arabic gum, guar gum and high methoxyl pectin. They also investigated the ability to deliver unstable high-value components (betacyanins and polyphenols). Guar gum increased both loading efficiency of bioactive components and antioxidant activity. Therefore, guar gum was reported to have the appropriate encapsulation properties in order to be used as an ingredient in functional foods.

Tian et al. [70] suggested using mixtures of polymers for target delivery because their combinations can fulfil more requirements than using only one polymer. Locust bean gum is non-ionic galactomannan. It belongs to the group of soluble fibers. In the food industry, locust bean gum is always used in combination with hydrocolloids because it will not gel on its own but enhances the gelation properties and strength of gels when added to the system [53]. Xanthan gum/guar gum and xanthan gum/locust bean gum were mixed with tea polyphenols and viscosity and polyphenol release rates were investigated. Systems containing xanthan gum/locust bean gum had higher viscosity. Generally, xanthan content had a positive impact on viscosity increase and release rates from this system were also lowered. It was concluded that diffusion rate of the bioactive molecules is strongly affected by the hydrogel composition [70].

Nanoemulsions encapsulated within hydrogel are also called “Trojan-horse” nanoparticles due to their ability to control the release of encapsulated particles in the gastrointestinal tract [71]. Demisli et al. [72] prepared nanoemulsion-based hydrogels for the delivery of curcumin, which belongs to the group of polyphenols. Curcumin is reported to have antimicrobial, anticancer and anti-inflammatory activity [53] but has low solubility and inferior target delivery; thus it is necessary to improve these poor characteristics by encapsulation. Nanoscale applications for delivering bioactive components have recently undergone a positive evolution, and it was proven that encapsulation at nanoscale is a viable approach for the improvement of these properties.

Investigation of phenolics stability during processing and interactions that occur in the food matrix are gaining in importance for food processors in order to determine the shelf life of gel products with putative health-promoting effects even though it is still largely unknown [66].

## 6. Retention of Aroma Compounds in Hydrogels

Food quality and its acceptability in the marketplace are determined by its organoleptic properties. Aroma compounds are delivered to gustatory and olfactory receptors after their release from the food matrix into the gas phase through an interface. Food products are usually not homogeneous systems and retention of aroma is often affected by carbohydrates, proteins, fats and other components present in the food matrix. Components of aroma can be physically entrapped within the matrix (so-called texture-specific effect) or bounded to the gelling agents of the matrix (so-called agent-specific effect). Nature, properties and amount of aroma compounds vary, thus affecting release and perception of overall aroma [73]. Diffusion mechanism types in the system have an impact on the release rate of aroma compounds from food systems. In the static fluid systems, mass transport is conducted by molecular diffusion and the aroma release rate is dependent on the resistance at the boundary layers and motions of the present molecules. Diffusion is responsible for the mass transport during the mechanical treatment and consequently, release rates are higher and aroma compounds are present in higher concentrations on the surface boundary layer [74].

Molecules responsible for the aroma of food systems are hydrocarbons, alcohols, ketones, aldehydes, acids and/or esters. Their molecular weights range between 100 and 250 g/mol [53]. In fruits, aroma is a very complex quality parameter and compounds which contribute most significantly to the fruit aroma are usually defined by their main properties: chemical structure, hydrophobicity and volatility. If added, hydrocolloids affect aroma of the system through two main mechanisms. One is due to the increased viscosity of the system which causes reduction of the diffusion rate in the media and the other is the binding of aroma molecules to hydrocolloids present in matrix. In order to investigate this phenomenon, Kopjar et al. [3] prepared hydrogels which contained two hydrocolloids (xanthan or guar gum) and two disaccharides (sucrose or trehalose). They studied the combined influence on retention of two aroma compounds (linalool and eugenol). Linalool is an acyclic monoterpene tertiary alcohol which is used as an aroma agent in the food and pharmaceutical industry. It originates from plants, members of *Lamiaceae*, *Lauraceae* and *Rutaceae* families [11]. Eugenol is another aroma component obtained from *Eugenia caryophyllata* buds and leaves. It is also used in pharmaceutical, cosmetics and food industry. Its derivatives are of great importance in medicine because they are used as anesthetics and antiseptics [75]. Both of these aroma compounds have several different positive effects on human health, such as antioxidant, antimicrobial, anti-inflammatory and anticancer activity [11,74]. In hydrogels prepared from xanthan and sucrose, retention of linalool increased with increasing xanthan concentration. In trehalose-containing hydrogels the opposite effect was observed, where hydrogels prepared with lower amounts of xanthan had better retention of linalool. The addition of guar gum improved the retention of linalool more than the addition of xanthan, with the exception of hydrogel that contained guar gum and trehalose. In general, retention of linalool increased with higher amounts of disaccharide added. Hydrogels with xanthan demonstrated a different behavior than those with guar gum due to the fact that xanthan and guar gum are different types of hydrocolloids. Xanthan gum is better water binder and it is anionic in nature, while guar gum is neutral. Retention of eugenol was also investigated. In samples with xanthan and both types of disaccharides, retention of eugenol decreased with the increase in the concentration of hydrocolloid. The opposite effect was observed in hydrogels containing guar gum and trehalose, where the increase in hydrocolloid concentration was associated with the increase of eugenol retention [3]. Zhang et al. [76] also concluded that solubility of the volatile aroma compounds can be affected by the addition of sugar to the solution. Hydrocolloids, disaccharides, water and their mutual interactions play an important role in the retention of linalool and eugenol. In xanthan solutions, ‘hydrophobic cavity’ is a phenomenon which is one of the possible mechanisms for capturing of aroma compounds. Also, prepared complexes can have different affinities for aroma compounds. Xanthan has higher water binding capacity and so it disturbs interactions with hydrophobic compounds such as linalool [3]. Overall retention is not affected by the nature of molecules but the composition of the system [77]. Also, Tromelin et al. [10] concluded that chemical groups are not the most important factors for the retention of aroma compounds, but the assembly of chemical properties. Although aroma is affected by viscosity, thickened systems with similar viscosity do not always have the same values of aroma retention. So, it can be concluded that binding of the components also affects aroma release [78].

Bylaite et al. [74] investigated the influence of xanthan on the release of aroma compounds. It was observed that limonene had the highest retention rate probably due to its hydrophobic character. Significant retention rates were also observed for esters and aldehydes. It was concluded that chemical structures and physicochemical characteristics of aroma compounds have a significant impact on their release behavior. For aldehydes and ketones, the same trend was observed. Hydrophobic structures (e.g., heptanal and octanal, ketones with higher carbon numbers) were also significantly retained, while alcohols were not retained in the system in the presence of xanthan. It was explained as “salting out” of hydrophilic compounds. In contrast with other hydrocolloids, xanthan has a more hydrophobic character and can create a hydrophobic enclosure in the molecules of aroma compounds. Finally, it was concluded that hydrocolloids can affect aroma compounds differently, not only due to their viscosity but also due to their polarity.

Misharina et al. [79] studied the effect of structure and composition of biopolymers (polysaccharides, animal protein gelatine and vegetable fibers) on the binding of aroma compounds. Investigated samples contained different organic compounds usually present in food products: aliphatic, mono- and bicyclic monoterpene hydrocarbons, sesquiterpenes, alcohols, ketones and acetates. It has been found that the inclusion of biopolymers lowers the aroma intensity and leads to its modification or disappearance. This phenomenon was the consequence of the reduction in diffusion of aroma compounds in combination with the increase of matrix viscosity. Also, certain interactions (e.g., formation of larger structures with lipids and polymers, complex formation and sorption on biopolymers, incorporation in microcavities) with food components caused weakening of aroma. They concluded that all biopolymers held monoterpene hydrocarbons. Bulk sesquiterpenes had the lower degree of binding and alcohols had the lowest binding rate. Aliphatic linalool and cyclic 4-terpineol had a similar sorption degree. Nevertheless, the substitution of the hydroxyl group in the side chain of α-terpineol caused the reduced sorption compared to 4-terpineol. Presence of the keto group in terpene limonene and cyclic ketone carvone caused a decrease of the binding factors. The adsorption of acetates to biopolymers was carried out more efficiently which is probably due to acetates being more hydrophobic than alcohols and ketones. Polar compounds, like linalool, are more soluble in water and they bond to polymers more efficiently than nonpolar compounds (e.g., limonene). In addition to polarity, structure and composition of polymers, their physicochemical condition and the presence of functional groups also affect the binding of aroma compounds. Citrus fibers bound monoterpene and sesquiterpene hydrocarbons and cyclic terpenyl acetate hydrocarbons better than alcohols and ketones. Compared to fibers, carboxymethylcellulose bound alcohols less efficiently. It had similar binding efficiency as maltodextrins with the exception of α-terpeniol (it was not bound to maltodextrine and was bound to carboxymethylcellulose). Evidently, the presence of carboxymethyl groups causes improvement of the sorption ability of carboxymethylcellulose compared to other glucose polymers. Xanthan did not bind α-terpineol (like starch and maltodextrin) and had the higher binding degree of aliphatic alcohols and ketones than starch. Guar gum had great efficiency in binding hydrocarbons and acetates and had the highest alcohol and ketones binding efficiency compared to other polymers. Sodium alginate adsorbed all compounds at a medium rate and was similar to maltodextrin, citrus fibers and xanthan gum binding rates. Although it contains polar groups in peptide chain, gelatine is a hydrophobic polymer. It bound acetates and mono- and sesquiterpenes hydrocarbons more efficiently and bound all ketones and alcohols (α-terpineol as well). It was observed that with the extension of the ester chains, their binding ability to gelatine increased, while polar alcohols like 3-hexanol did not bind successfully. Finally, gum arabic and guar gum had maximal binding ability of polar and nonpolar compounds [79]. In Table 1, there are examples of other edible polymers and/or their combination used for preparation of hydrogels in which retention of listed aromas was investigated.

## 7. Conclusions

Although phenolic and aroma compounds are most often unstable during food manufacturing and thus their delivery to target sites through food is disturbed, reliable ways for their preservation do exist. In the present review, possibilities of using hydrogels as phenolic and aroma compound carriers are discussed. Proper formulation of both natural and synthetic hydrogels is of extreme importance in order to achieve the retention of high-valuable bioactive compounds. Further, it is necessary to know their physicochemical properties and properties of the polymer used for the hydrogel preparation. Presently, there are numerous applications of hydrogels in the pharmaceutical, cosmetic and food industries. Due to the progress made in their development, it is possible to obtain gels, particles and edible films at nanoscale. However, the main objective is to produce hydrogels which are safe for consumption, while at the same time their production needs to be profitable. In the future there will be an emphasis on development of the “smart hydrogels”, which will be capable of concurrently carrying out the target delivery and preservation of encapsulated compounds. In order to improve the knowledge base related to this area and to obtain hydrogels with desired characteristics, whose application in food industry would also be cost-effective, further investigations are required.

## Figures and Tables

**Table 1:** Overview of edible polymers used for preparation of hydrogels with the aim of investigating retention of aroma compounds.

Aroma Compounds	Type of Hydrocolloid Used for Hydrogel Preparation	Conditions for the Hydrogel Preparation	Reference
ESTERS			
Benzyl acetate	Pectin, gelatine	Dissolving hydrocolloids at 60 °C for 2 min	[80]
Butyl acetate	Pectin, starch, gelatine	Dissolving hydrocolloids at 80 °C for 5 min	[76]
	Pectin	Dissolving hydrocolloid while boiling for 2 min	[29]
Butyl pentanoate	Pectin, carrageenan	Dissolving hydrocolloids (temperature and time not specified)	[14]
Ethyl acetate	Pectin, starch, gelatine	Dissolving hydrocolloids at 60 °C for 2 min or at	[13]
		80 °C for 5 min	[76]
	Pectin	Dissolving hydrocolloid while boiling for 2 min	[29]
Ethyl butanoate	Pectin, starch, gelatine	Dissolving hydrocolloids at 80 °C for 5 min	[76]
Ethyl trans-2-butenoate	Pectin, carrageenan	Dissolving hydrocolloids (temperature and time not specified)	[14]
Ethyl butyrate	Starch and/or carrageenan	Dissolving hydrocolloids at 85 °C for 10 min	[77]
	Pectin, starch, gelatine	Dissolving hydrocolloids at 60 °C for 2 min	[13]
	Pectin, gelatine	Dissolving hydrocolloids at 60 °C for 2 min	[80]
	Pectin	Dissolving hydrocolloid while boiling for 2 min	[28]
Ethyl heptanoate	Pectin, carrageenan	Dissolving hydrocolloids (temperature and time not specified)	[14]
Ethyl hexanoate	Starch and/or carrageenan	Dissolving hydrocolloids at 85 °C for 10 min	[77]
		or at 90 °C for 30 min	[78]
	Pectin, starch, gelatine	Dissolving hydrocolloids at 80 °C for 5 min	[76]
	Pectin, gelatine	Dissolving hydrocolloids at 60 °C for 2 min	[80]
	Pectin	Dissolving hydrocolloids while boiling for 2 min	[28,29]
	Pectin, carrageenan	Dissolving hydrocolloids (temperature and time not specified)	[14]
Ethyl iso-pentanoate	Pectin, gelatine	Dissolving hydrocolloids at 60 °C for 2 min	[80]
Ethyl propanoate	Pectin, carrageenan	Dissolving hydrocolloids (temperature and time not specified)	[14]
cis-3-Hexenyl acetate	Pectin, gelatine	Dissolving hydrocolloids at 60 °C for 2 min	[80]
	Pectin	Dissolving hydrocolloids while boiling for 2 min	[28]
Isoamyl acetate	Starch and/or carrageenan	Dissolving hydrocolloids at 90 °C for 30 min	[78]
	Pectin, carrageenan	Dissolving hydrocolloids (temperature and time not specified)	[14]
Isopentyl acetate	Pectin	Dissolving hydrocolloids while boiling for 2 min	[28]
Isopropyl 2-methyl-2-butenoate	Pectin, carrageenan	Dissolving hydrocolloids (temperature and time not specified)	[14]
Isopropyl butanoate	Pectin, starch, gelatine	Dissolving hydrocolloids at 80 °C for 5 min	[76]
Methyl anthranilate	Pectin, gelatine	Dissolving hydrocolloids at 60 °C for 2 min	[80]
Methyl butanoate	Pectin, starch, gelatine	Dissolving hydrocolloids at 80 °C for 5 min	[76]
Methyl cinnamate	Starch and/or carrageenan	Dissolving hydrocolloids at 85 °C for 10 min	[77]
Methyl hexanoate	Pectin, starch, gelatine	Dissolving hydrocolloids at 80 °C for 5 min	[76]
Propyl acetate	Pectin	Dissolving hydrocolloid while boiling for 2 min	[29]
Styrallyl acetate	Pectin, gelatine	Dissolving hydrocolloids at 60 °C for 2 min	[80]
ALCOHOLS			
1-Butanol	Pectin, starch, gelatine	Dissolving hydrocolloids at 60 °C for 2 min	[13]
	Pectin	Dissolving hydrocolloids while boiling for 2 min	[29]
3-Methyl-1-butanol	Pectin, starch, gelatine	Dissolving hydrocolloids at 60 °C for 2 min	[13]
	Pectin	Dissolving hydrocolloids while boiling for 2 min	[29]
1-Hexanol	Pectin, starch, gelatine	Dissolving hydrocolloids at 80 °C for 5 min	[76]
	Pectin	Dissolving hydrocolloids while boiling for 2 min	[29]
cis-3-Hexenol	Pectin, gelatine	Dissolving hydrocolloids at 60 °C for 2 min	[80]
	Pectin	Dissolving hydrocolloids while boiling for 2 min	[28,29]
	Starch and/or carrageenan	Dissolving hydrocolloids at 85 °C for 10 min	[77]
Linalool	Starch and/or carrageenan	Dissolving hydrocolloids at 85 °C for 10 min	[77]
	Pectin, starch, gelatine	Dissolving hydrocolloids at 80 °C for 5 min	[76]
	Pectin	Dissolving hydrocolloids while boiling for 2 min	[28]
Nerolidol	Pectin, starch, gelatine	Dissolving hydrocolloids at 80 °C for 5 min	[76]
2-Nonanol	Pectin	Dissolving hydrocolloids while boiling for 2 min	[29]
2-Pentanol	Pectin	Dissolving hydrocolloids while boiling for 2 min	[29]
1-Propanol	Pectin	Dissolving hydrocolloids while boiling for 2 min	[29]
ALDEHYDES			
2-Ethylbutanal	Pectin, carrageenan	Dissolving hydrocolloids (temperature and time not specified)	[14]
trans-2-Methyl-2-butenal	Pectin, carrageenan	Dissolving hydrocolloids (temperature and time not specified)	[14]
Heptanal	Pectin, starch, gelatine	Dissolving hydrocolloids at 60 °C for 2 min	[13]
	Pectin	Dissolving hydrocolloids while boiling for 2 min	[29]
Hexanal	Pectin, starch, gelatine	Dissolving hydrocolloids at 60 °C for 2 min	[13]
		or at 80 °C for 5 min	[76]
	Pectin, gelatine	Dissolving hydrocolloids at 60 °C for 2 min	[80]
	Pectin	Dissolving hydrocolloids while boiling for 2 min	[28,29]
(E)-2-hexenal	Pectin, starch, gelatine	Dissolving hydrocolloids at 80 °C for 5 min	[76]
Octanal	Pectin	Dissolving hydrocolloids while boiling for 2 min	[29]
Vanillin	Starch and/or carrageenan	Dissolving hydrocolloids at 85 °C for 10 min	[77]
KETONES			
2-Butanone	Pectin, starch, gelatine	Dissolving hydrocolloids at 60 °C for 2 min	[13]
	Pectin	Dissolving hydrocolloids while boiling for 2 min	[29]
2,3-Butanedione	Pectin	Dissolving hydrocolloids while boiling for 2 min	[29]
Gamma-Decalactone	Starch and/or carrageenan	Dissolving hydrocolloids at 85 °C for 10 min	[77]
2-Decanone	Pectin, starch, gelatine	Dissolving hydrocolloids at 60 °C for 2 min	[13]
	Pectin	Dissolving hydrocolloids while boiling for 2 min	[29]
Diacetyl	Pectin, starch, gelatine	Dissolving hydrocolloids at 60 °C for 2 min	[13]
2-Heptanone	Pectin, starch, gelatine	Dissolving hydrocolloids at 60 °C for 2 min	[13]
	Pectin	Dissolving hydrocolloids while boiling for 2 min	[29]
		Dissolving hydrocolloids (temperature and time not specified)	[14]
2,6-Dimethylheptan-4-one	Pectin, carrageenan	Dissolving hydrocolloids (temperature and time not specified)	[14]
4-Methylpent-3-en-2-one	Pectin, carrageenan	Dissolving hydrocolloids (temperature and time not specified)	[14]
4-Methylpentan-2-one	Pectin, carrageenan	Dissolving hydrocolloids (temperature and time not specified)	[14]
β-Ionone	Pectin, gelatine	Dissolving hydrocolloids at 60 °C for 2 min	[80]
2-Octanone	Pectin, starch, gelatine	Dissolving hydrocolloids at 60 °C for 2 min	[13]
	Pectin	Dissolving hydrocolloids while boiling for 2 min	[29]
ACIDS			
Acetic acid	Pectin, starch, gelatine	Dissolving hydrocolloids at 80 °C for 5 min	[76]
Hexanoic acid	Pectin, starch, gelatine	Dissolving hydrocolloids at 80 °C for 5 min	[76]
2-Methylbutanoic acid	Pectin, starch, gelatine	Dissolving hydrocolloids at 80 °C for 5 min	[76]
Propanoic acid	Pectin, starch, gelatine	Dissolving hydrocolloids at 80 °C for 5 min	[76]
TERPENES			
Limonene	Pectin	Dissolving hydrocolloids while boiling for 2 min	[28]
		or for 20 min	[81]
Menthone	Pectin	Dissolving hydrocolloids while boiling for 2 min	[28]
